# Supplementary material for: Longwave-transparent low-emissivity material
Source: Sci Adv. 2026 Mar 6;12(10):eaeb8872. doi: 10.1126/sciadv.aeb8872 (PMC12965322; doi:10.1126/sciadv.aeb8872)
Supplement: Supplementary file 1 — Supplementary Text S1 to S8 Figs. S1 to S23 Legends for movies S1 to S3 References [file sciadv.aeb8872_sm.pdf]

Science Advances
AAAS

Supplementary Materials for

Longwave-transparent low-emissivity material

Yue Zhang *et al.*

Corresponding author: Longnan Li, longnanli@ciomp.ac.cn; Tie Jun Cui, tjcui@seu.edu.cn;
Wei Li, weili1@ciomp.ac.cn

Sci. Adv. **12**, eaeb8872 (2026)
DOI: 10.1126/sciadv.aeb8872

The PDF file includes:

Supplementary Text S1 to S8
Figs. S1 to S23
Legends for movies S1 to S3
References

Other Supplementary Material for this manuscript includes the following:

Movies S1 to S3

Supplementary Text

Supplementary text 1. Analysis of scattering efficiency of NaCl micro-nanoparticles

Mie theory provides exact analytical solutions for the light intensity angle distribution of spherical particles with arbitrary composition and diameter. Optical simulations were conducted using the Finite-Difference Time-Domain (FDTD) method (FDTD Solution 2020 R2, Lumerical). The scattering efficiency, defined as the scattering cross-section normalized by the geometric area, was calculated under a normally incident total field scattering field (2-25 μm) combined with Perfectly Matched Layer (PML) boundaries. The optical power cross-section was used to determine microparticle cross-sections. Frequency domain field profile monitors were employed to obtain the electromagnetic field distribution. The refractive index and extinction coefficient of NaCl used in our analysis are taken from tabulated literature data (*51,52*) over the visible–mid-infrared range and are plotted in Fig. 2A-i.

Supplementary text 2. Environmental stability and mechanical robustness

To evaluate the environmental stability of the LLM film, we conducted a series of tests. The LLM film was stored in an indoor environment for 40 days and 90 days, after which its infrared spectrum was measured. As shown in Supplementary Fig. S9A, the average infrared reflectance of the original LLM film was 80.16%. After 40 days, the average infrared reflectance slightly decreased to 79.86%, representing a 0.3% reduction. The relative humidity during the test was maintained at 46±5%. After 90 days, the average infrared reflectance decreased to 77.36%, representing a 2.80% reduction. The relative humidity during the test was maintained at 64±5%. To test the durability of the LLM in a high-humidity environment, we placed the LLM in an environment with a humidity of 90±5% and continuously humidified it for 36 hours. As shown in Supplementary Fig. S9B, the average infrared reflectance of the original LLM film was 82.13%. After high-humidity test, the average infrared reflectance decreased to 76.24%, representing a 5.89% reduction.

In a separate test, a sample consisting of NaCl microparticles encapsulated in a non-porous PE film (infrared transparent) was fully submerged in deionized water for 24 hours. The infrared reflectance spectrum was measured before and after the immersion. The results, shown in Fig. S9C, indicate only a minor decrease in reflectance, demonstrating that the optical functionality of the scattering core is largely preserved even under direct water exposure, highlighting the effectiveness of polymer-based encapsulation in protecting the hygroscopic salt.

Finally, a strip of the standard NanoPE-encapsulated LLM film was subjected to a bending endurance test. The film was bent to approximately 180 degrees and then released back to its flat state, and this cycle was repeated 15 times. Visual inspection (Fig. S9D) confirmed that the film surface and structure remained intact, with no observable cracks, delamination, or particle shedding. This test confirms the film's ability to withstand mechanical stresses encountered during installation and use in flexible applications.

Supplementary Text 3. Cost Estimation of LLM Film

A preliminary direct material cost analysis estimates the production cost at approximately ¥6 per square meter. The cost structure is as follows: NaCl (883 g, ¥ 1.32), SEBS binder (77 g, ¥ 1.93), THF solvent assuming 90% recovery (88 g loss, ¥ 1.32), and two layers of nanoPE substrate (¥ 1.50). This cost is highly competitive with common metallic low-e radiant barriers (e.g., the price of reflective commercial insulation wall sticker used in this study is ~ ¥ 12). Furthermore, sourcing NaCl from desalination brine by-products could further reduce cost. This favorable cost

structure, combined with the unique value of longwave transparency, strongly supports the scalability and practical feasibility of LLM for real-world applications.

Supplementary text 4. Transmittance spectra in GHz-THz

The transmittance spectra in the 1-40 GHz range were calculated by converting the S parameters. The S parameter is crucial in microwave transmission, where S_{21} represents the insertion loss and indicates how much energy is transmitted to the receiver. Using the vector network analyzer and the appropriate frequency antenna, both the samples and the control groups were tested, and the corresponding S parameters were obtained.:

$$S_{21} = 20\log_{10}\left|\frac{V_{trans}}{V_{inc}}\right| \quad (dB) \tag{S1}$$

The relationship between S_{21} and transmittance is (*67*):

$$T = |S_{21}|^2 = {S_{21\_r}}^2 + {S_{21\_i}}^2 \tag{S2}$$

Supplementary text 5. Evaluation of thermal insulation performance

To investigate the influence of the thermal radiation characteristics of LLM on building heating energy consumption, we constructed an experimental model simulating building heating in winter and tested it in a cold outdoor environment, as shown in Fig. 3A. During the experiment, we measured the real-time temperature inside the box (T_{in}) and the real-time heating voltage (U) and current (I) of the electric heater. Heating energy consumption was calculated for different insulation layers while maintaining a consistent internal temperature. The formula for calculating heating energy consumption is as follows:

$$W = Pt = UIt \tag{S3}$$

To demonstrate the thermal stability of LLM film regarding temperature variation, we placed a box covered with the thermal insulation layer on both the outside and inside in an outdoor environment. As shown in Fig. 3G, we recorded the real-time temperature curve and calculated the variance of the real-time temperature over a two-day period to evaluate the thermal insulation performance of the LLM. The variance of the inner temperature is calculated as follows:

$$S^2 = \frac{1}{n}\sum_{i=1}^{n}(Tin_i - T_{avg})^2 \tag{S4}$$

Supplementary text 6. Model development for energy saving

We used EnergyPlus (version 9.6) to perform energy consumption simulations on commercial reference building models (post-1980 midrise apartments, as defined by the US Department of Energy) for 16 cities: Miami, Houston, Phoenix, Atlanta, Los Angeles, Las Vegas, San Francisco, Baltimore, Albuquerque, Seattle, Chicago, Boulder, Minneapolis, Helena, Duluth, and Fairbanks. The model building is a four-story rectangular structure consisting of 31 apartments and an office, with a length-to-width ratio of 2.74 (16.91 m width, 46.33 m length, and 12.19 m height). The total floor area is 3135 m^2, with windows accounting for 15% of the total wall area.

The HVAC system of the building uses DX cooling (COP = 3.13) for cooling and gas furnaces (burner efficiency = 0.8) and electric heaters (efficiency = 1) for heating. The efficiency of the fans is 0.536. The indoor air temperature is set at a constant 22°C, and hourly weather data (TMY2) for the different cities are used for external weather conditions. The U-Factor of the windows in the building model is 6.927, and the solar heat gain coefficient is 0.25.

To calculate the HVAC energy consumption of the building using low-e windows, we set the solar transmittance of the windows to 0.8 and the infrared emissivity to 0.1. To evaluate the HVAC energy consumption of the building using low-emissivity materials, we used experimental data

(solar absorptance: 0.02, MIR emissivity: 0.15, which is the average measured solar and MIR emissivity of the LLM film) to modify the optical properties of the interior walls, exterior walls, roofs, and floor surfaces of the building.

Simulations were conducted to analyze the energy usage of the original building model, the building model with low-e windows, and the building model using low-e materials throughout the entire structure (windows, walls, roofs, and floors). The annual energy-saving values were normalized by dividing them by the total floor area of the simulated building. The annual energy savings for the total HVAC system, heating, cooling, and fans are shown in Supplementary Fig. S16.

Supplementary text 7. Constellation diagram in MM-Wave video transfer

Digital modulation during MM-Wave video data transfer was characterized by a constellation diagram, which defines two fundamental parameters: signal distribution and the mapping relationship with modulated digital bits. The relationship between a constellation point and the transmitted bit sequence is known as mapping. A modulation technique is fully described by its signal distribution and mapping, encapsulated in the constellation diagram. The experimental communication system uses QPSK modulation, with the QPSK signal
S_i(t) expressed as:

$$Si(t) = Acos(\omega t + \theta i) \quad i = 1,2,3,4 \quad 0 < t < Ts \qquad \text{(S5)}$$

where Ts denotes the symbol interval, and θi represents the phase of a sinusoidal carrier: θi can be π/4, 3π/4, 5π/4, 7π/4, corresponding to four different phase states, typically arranged in a square or cross pattern on a two-dimensional coordinate system(*68*). Each point corresponds to a specific signal point can be identified and decoded at the receiver, allowing for the accurate recovery of transmitted data.

Supplementary text 8. Wireless charging and wireless doorbell tests

To demonstrate the potential of LLM in intelligent life scenarios, we designed wireless charging and wireless doorbell tests. In the wireless charging scenario (as shown in Supplementary Fig. S23 and Supplementary Movie S2), the phone (P40 Pro, HUAWEI) used supported wireless charging with a maximum charging power of 27W and a charging frequency of 110-205 kHz. When the phone was placed directly on the wireless charger (W081, HUAWEI), it charged normally, and the phone screen displayed a charging power of 27W. When LLM was placed between the phone and the wireless charger, it still charged wirelessly with a power of 27W. However, when aluminum foil, a metal low-e material, was placed between the phone and the wireless charger, the phone could not be charged.

In the wireless doorbell experiment (as shown in Supplementary Movie S3), pressing the doorbell switch transmitted a wireless signal to the doorbell at a frequency of 447 MHz, causing it to ring. To simulate the application of a low-e insulation layer on the roof, walls, and floor of a house, we lined the six inner walls of a foam box with either LLM or aluminum foil. When the wireless doorbell was placed inside the foam box lined with LLM, pressing the doorbell switch outside the box caused the doorbell to ring inside, demonstrating that the wireless signal penetrated the LLM insulation layer. Conversely, when the wireless doorbell was placed inside the foam box lined with aluminum foil, pressing the doorbell switch outside the box did not cause the doorbell to ring, proving that the wireless signal was shielded by the aluminum foil and could not penetrate the box.

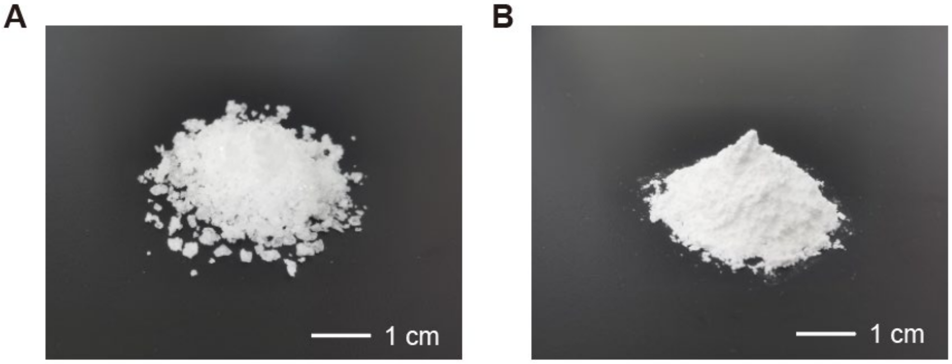

Fig. S1. Photographs of raw and processed NaCl particles. (A) Raw NaCl crystals. (B) Processed NaCl microparticles.

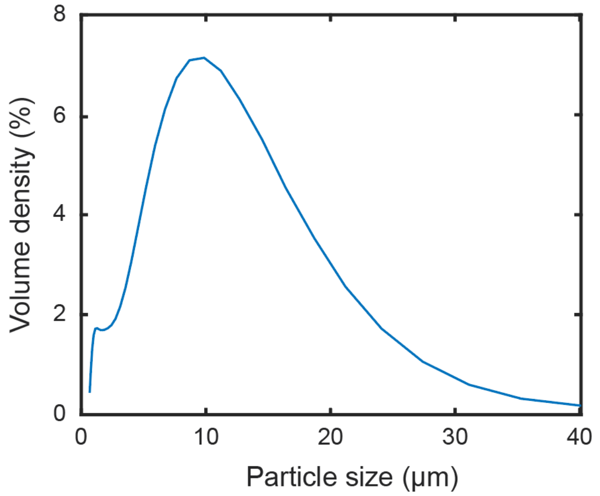

Fig. S2. Size distribution of NaCl micro-nanoparticles. Histogram showing the diameter distribution of particles used to construct the LLM.

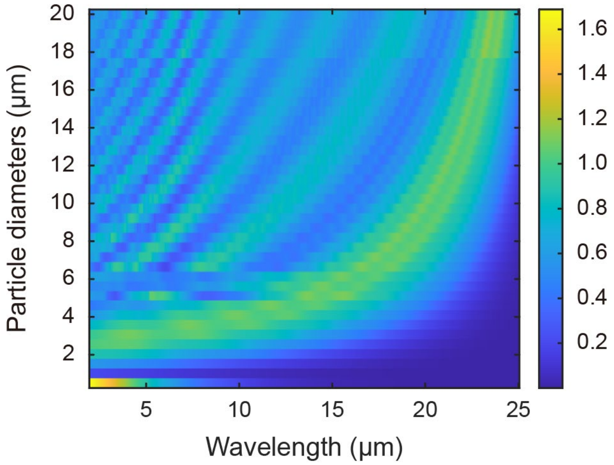

Fig. S3. Scattering efficiency of single NaCl particles. Calculated scattering efficiency for NaCl particles of various diameters in the mid-infrared range.

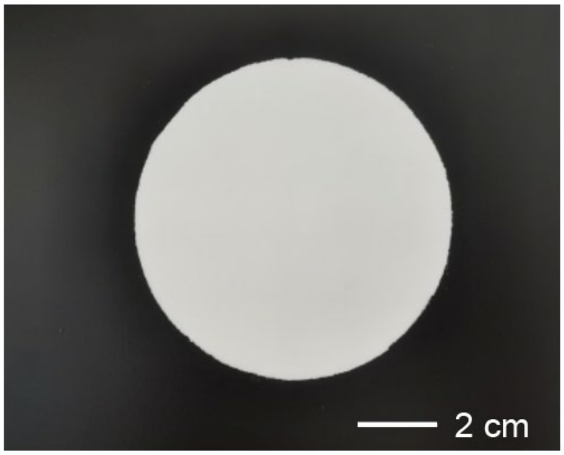

Fig. S4. Photograph image of pure LLM sample. The sample was fabricated by NaCl microparticles through high temperature sintering process.

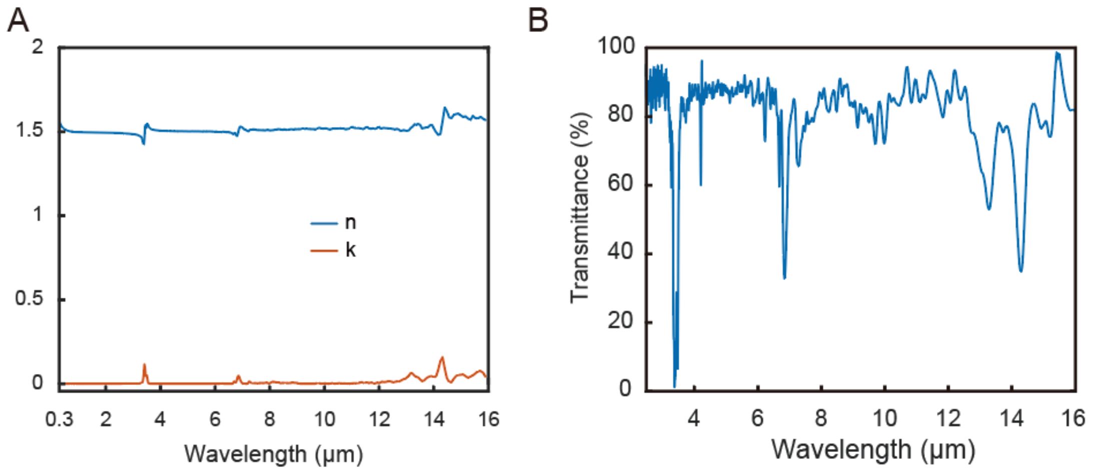

Fig. S5. Opticla properties of SEBS film. (A) The complex spectral refractive index (*n* + i*k*) of SEBS in the 0.3-16 μm range. (B) Spectral MIR reflectance of SEBS. The SEBS film for optical property characterization was prepared by mixing SEBS powder with THF and then spin coating. SEBS exhibits a high average transmittance of 0.823 in the 2.5-13 μm range. SEBS is chosen as the binder for LLM due to its high transmittance and low absorption in the infrared spectrum.

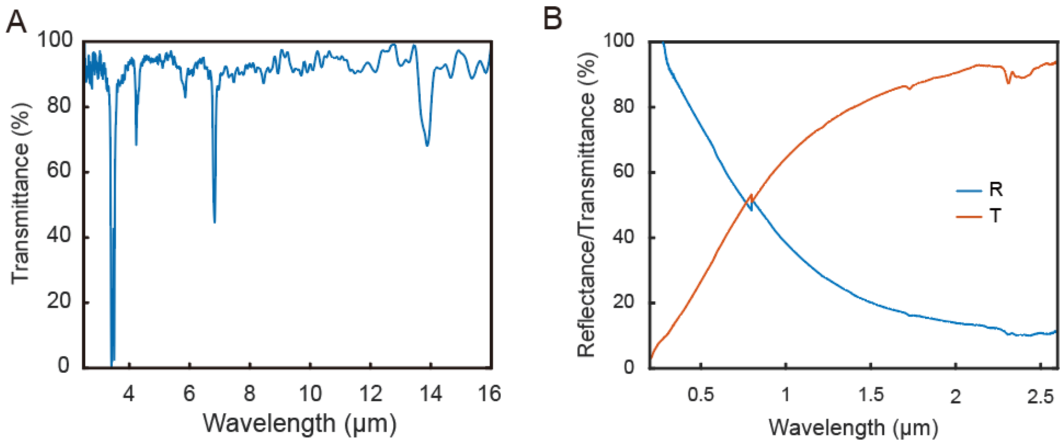

Fig. S6. Optical properties of NanoPE film. (A) spectral infrared reflectance and (B) spectral reflectance and transmission in the visible and near-infrared (NIR) wavelength ranges of the NanoPE film. The NanoPE film exhibits a high average transmittance of 0.909 in the 2.5-14 µm range.

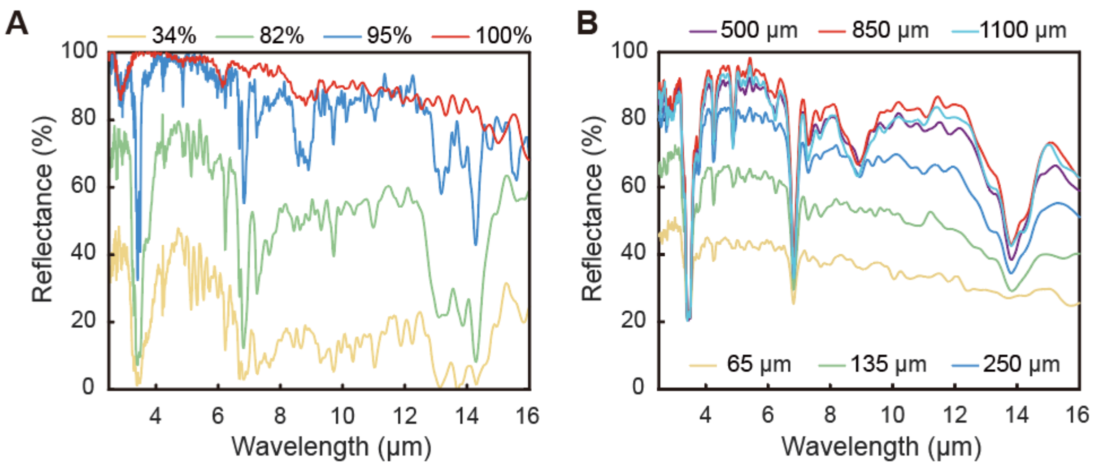

Fig. S7. Effect of composition and thickness on LLM reflectance. (A) Spectral reflectance of LLM in the mid-infrared (MIR) range (2.5 - 16μm) varying NaCl ratios of 34%, 82%, 95%, and 100% respectively. (B) Spectral reflectance of LLM film in the MIR range for different film thicknesses ranging from 65 μm to 1100 μm.

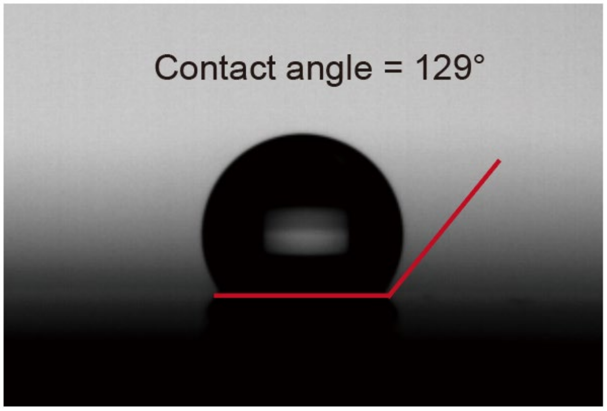

Fig. S8. Water contact angle of the LLM surface. The NanoPE-encapsulated LLM surface shows a contact angle of is 129°, demonstrating its considerable hydrophobicity, pollution resistance, and durability.

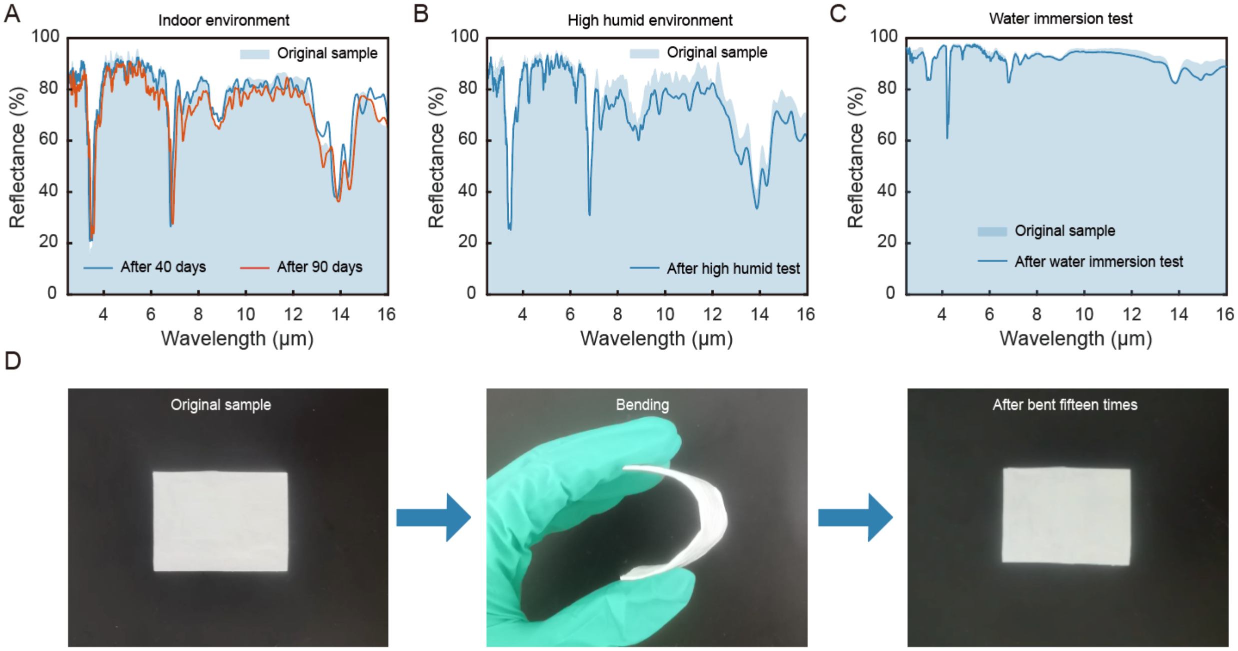

Fig. S9. Durability test and bending endurance test of the LLM. (A) Spectral infrared reflectance of LLM placed indoors after 40 days and 90 days. After 40 days, the average infrared reflectance decreased by 0.3% (Relative humidity: 46±5%). After 90 days, the average infrared reflectance decreased by 2.8% (Relative humidity: 64±5%). (B) Spectral infrared reflectance of LLM placed in high humid environment. After high humid test, the average infrared reflectance decreased by 5.89% (Relative humidity: 90±5%, humidified for 36 hours). (C) Spectral infrared reflectance of PE- encapsulated LLM placed in water for 24 hours. After water immersion test, the average infrared reflectance decreased by 1.3%. (D) Visual imaging of LLM film surface during repeated bending endurance test.

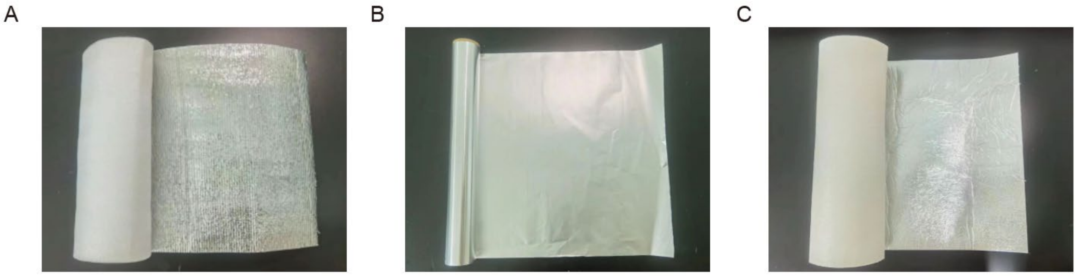

Fig. S10. Photographs of common reflective insulation materials. (A) Commercial insulation film, (B) aluminum foil, and (C) commercial insulation wall sticker.

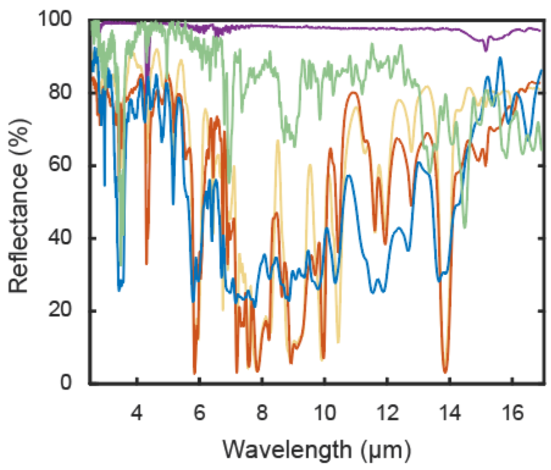

Fig. S11. Optical properties of control group. Spectral infrared reflectance of LLM (green), aluminum foil (purple), commercial insulation film (red), commercial insulation wall sticker (blue) and commercial bubble insulation film (yellow). The average infrared reflectance of the control groups are: 85.2% (LLM), 99.3% (aluminum foil), 55.8% (commercial insulation film), 45.5% (commercial insulation wall sticker) and 60.3% (commercial bubble insulation film).

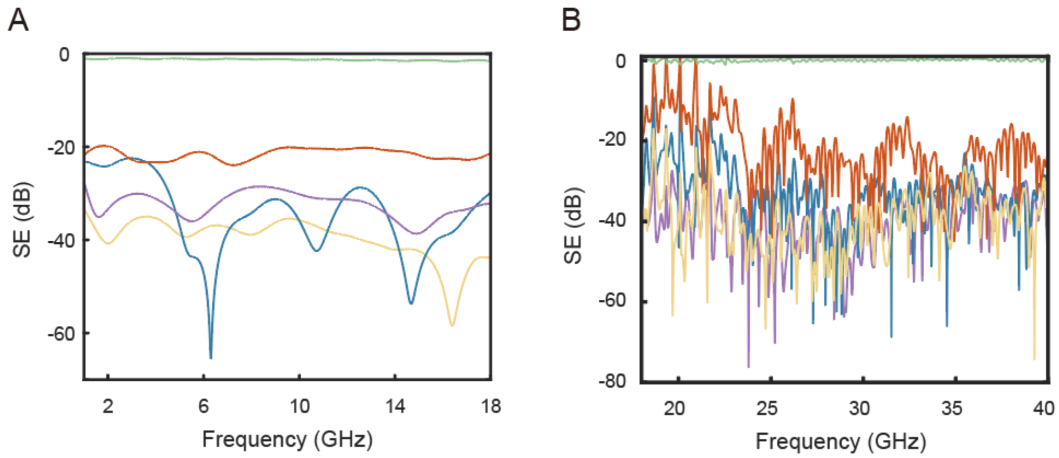

Fig. S12. Electromagnetic shielding effectiveness of different materials. Measured effectiveness of electromagnetic shielding of LLM (green), aluminum foil (purple), commercial insulation film (red), commercial insulation wall sticker (blue) and commercial bubble insulation film (yellow). (A) 1-18GHz. (B) 18-40GHz. The electromagnetic shielding effect of LLM is close to 0, which means that it has high transmittance and no shielding effect on the electromagnetic wave of 1-40GHz. The SE of other insulation materials with metal components is larger than 20dB, which indicates that it has strong shielding effect on electromagnetic waves, hindering the transmission of information and energy.

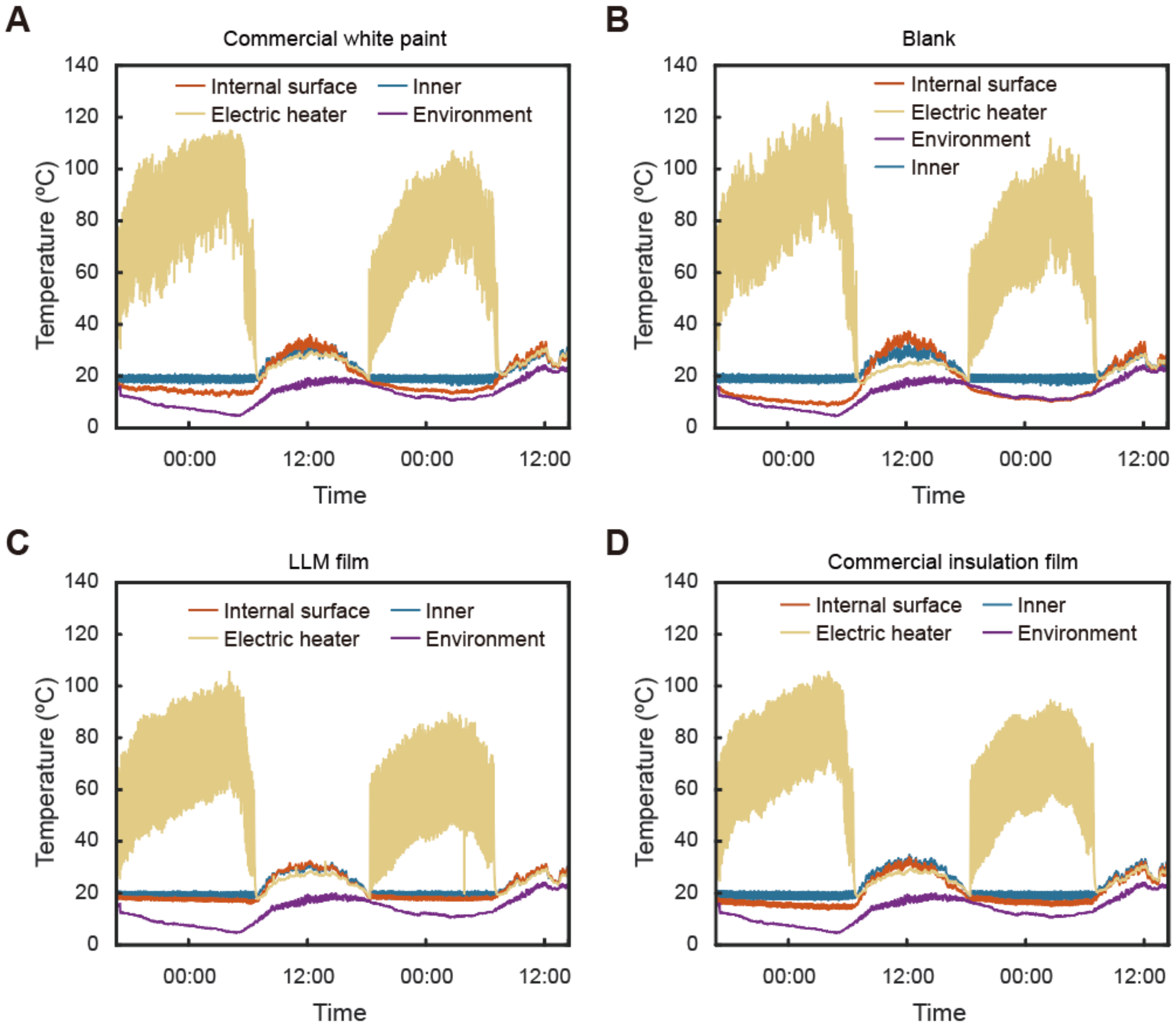

Fig. S13. Real-time temperature profiles during outdoor heating tests. Internal surface, interior, heater, and ambient temperatures for boxes covered with (A) white paint, (B) no cover (blank), (C) LLM film, and (D) commercial insulation film. On cold nights, the temperature of the box covered by LLM films was lower than that of the control groups, respectively, while the temperature of the inner surface of the box was higher and more stable than that of the control groups.

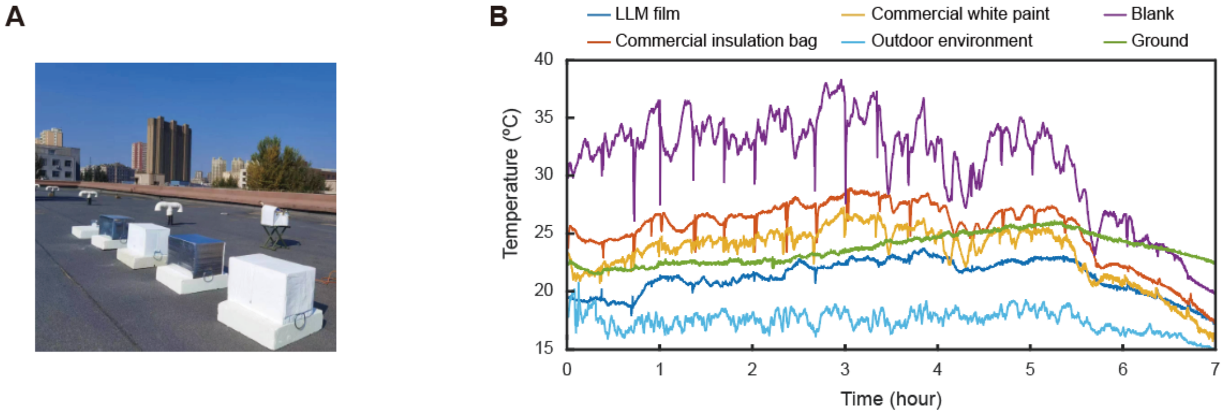

Fig. S14. Outdoor cooling performance. (A) Photograph image of the heat gain test setup of the cold storage simulant box with ice cubes inside at hot outdoor environment. (B) Measured inner real-time temperature curves with different surfaces in the box.

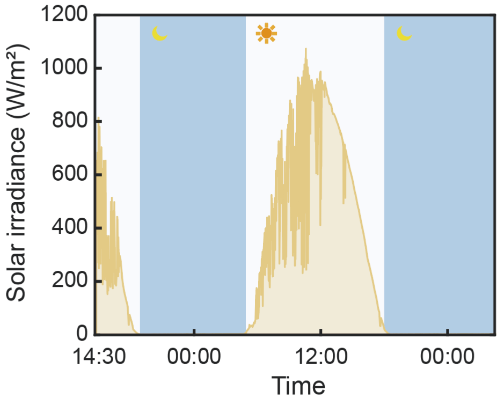

Fig. S15. Solar irradiance during outdoor thermal stability test.

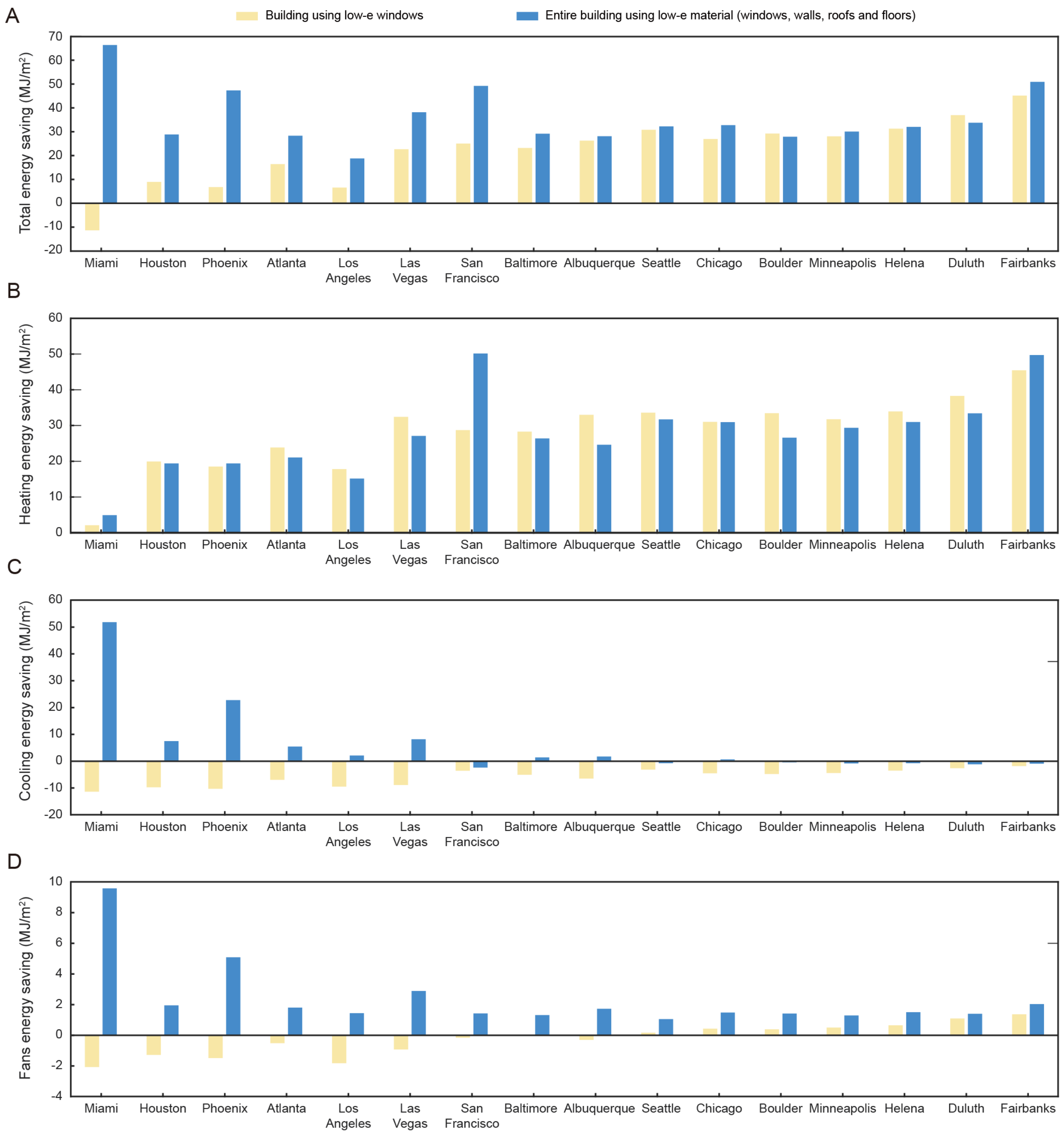

Fig. S16. Simulated annual HVAC energy savings. Energy savings for a typical midrise apartment building in different climate zones across the United States, using low-e windows (yellow) and low-e materials throughout the entire building, including windows, walls, roofs, and floors (blue). (A) Total energy savings. (B) Heating energy savings. (C) Cooling energy savings. (D) Fan energy savings.

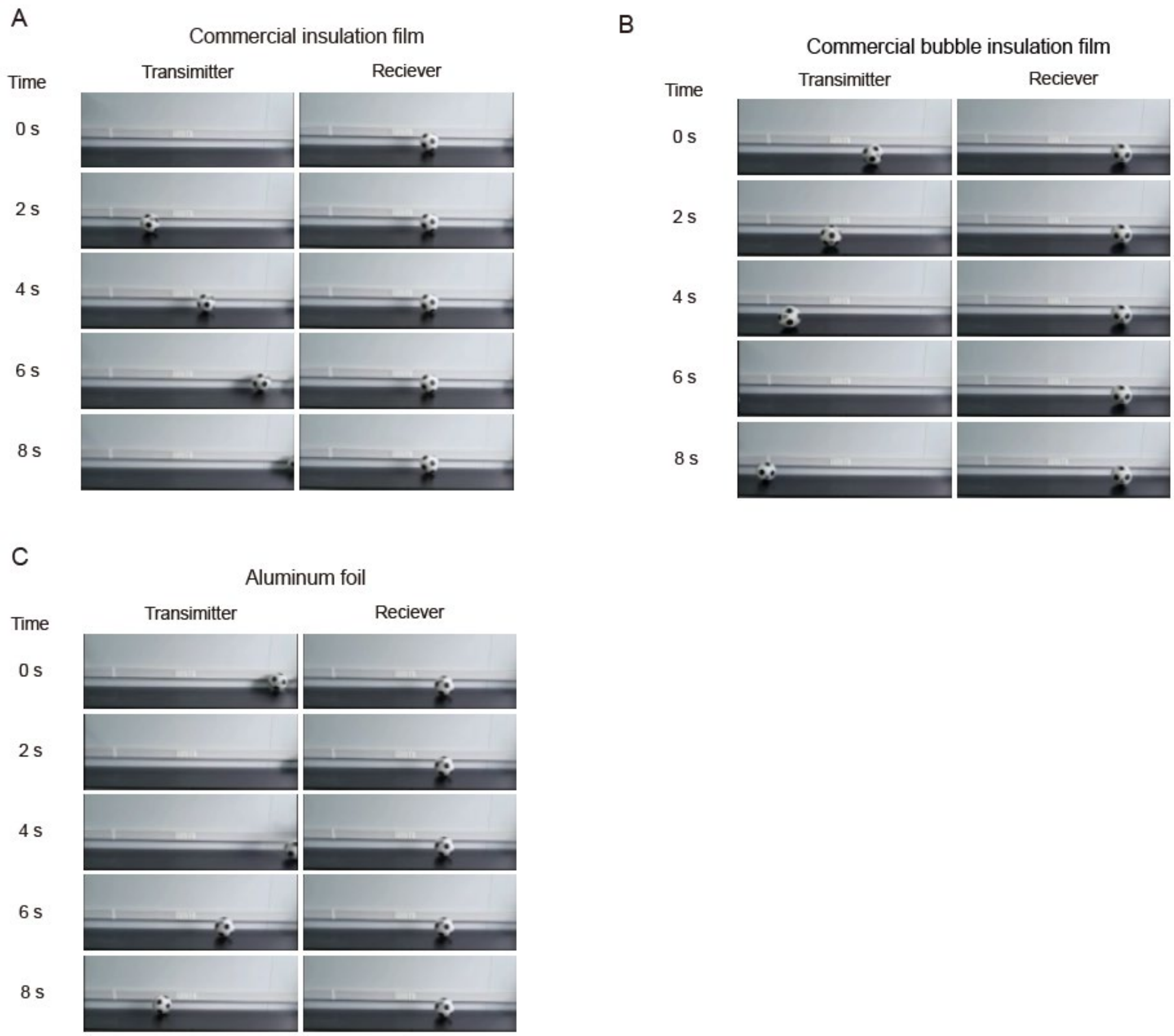

Fig. S17. Video-transmission quality through different insulation barriers. Time-series screenshots of the transmitted and received video. When using (A) commercial insulation film, (B) commercial bubble insulation film and (C) aluminum foil as building insulation layer, the video signal transmission failed. The video received by receiver is stuck and no longer played, corresponding to the position of the ball on the screenshots unchanged.

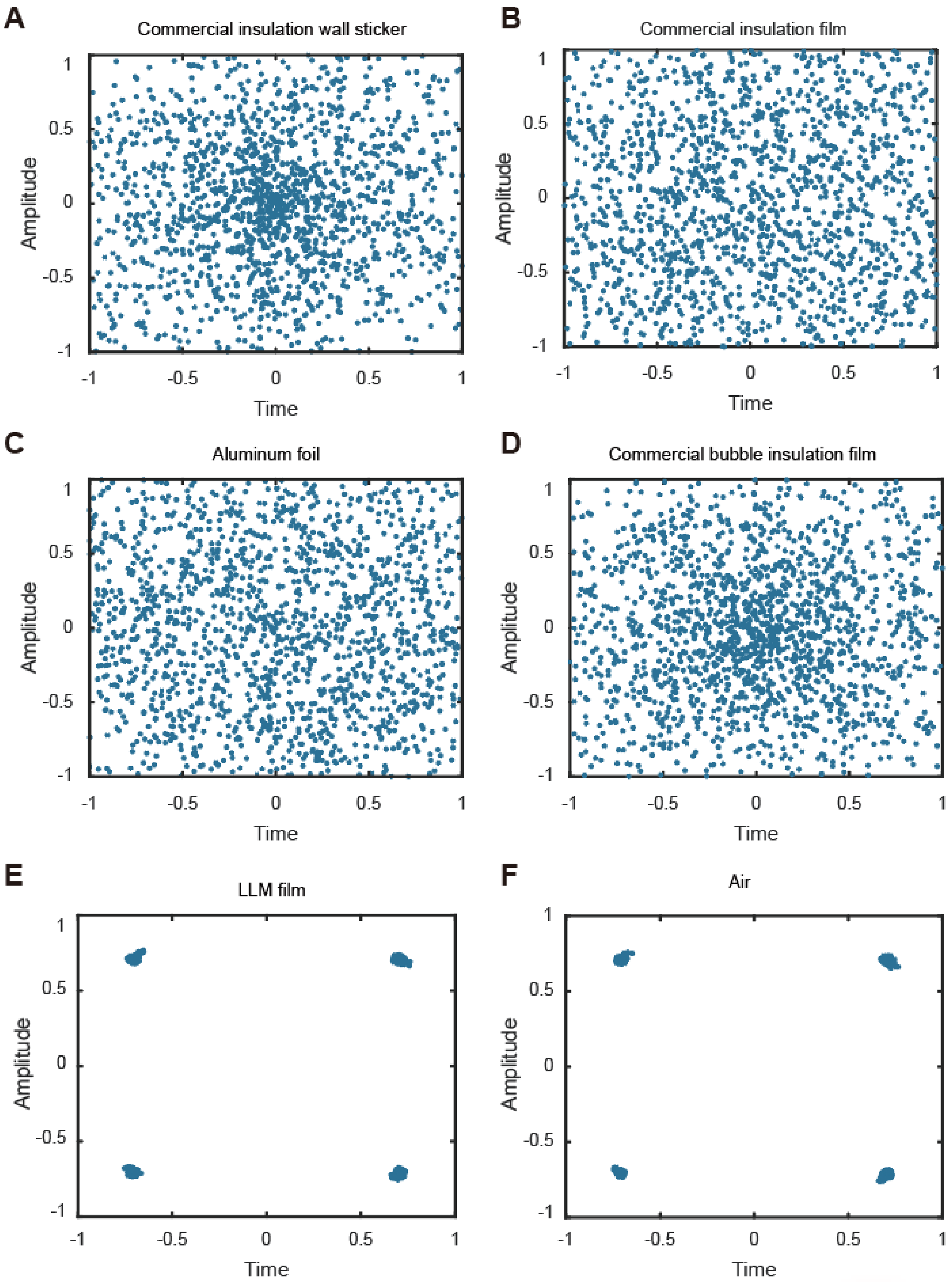

Fig. S18. Constellation diagrams for mm-wave communication through various materials. Diagrams obtained when using (A) commercial wall sticker, (B) commercial insulation film, (C) aluminum foil, (D) commercial bubble film, (E) LLM film, and (F) air (reference). Only LLM and air allow clear, stable constellation points.When using commercial wall stickers,

commercial insulation film, and aluminum foil as building insulation layers, the communication link was blocked, preventing the signal energy from reaching the receiver. The received signal energy was lower than the noise level, resulting in a disordered constellation diagram. Consequently, the receiver could not correctly demodulate the transmitted bit information, causing the communication system to fail and the video to be unplayable. In contrast, when using LLM films as the building layer, the communication link was not blocked. The transmitted signal energy reached the receiver, resulting in a high signal-to-noise ratio. The constellation diagram points were scattered and stable, allowing the receiver to correctly demodulate the transmitted bit information. As a result, the communication system functioned normally, and the video played successfully.

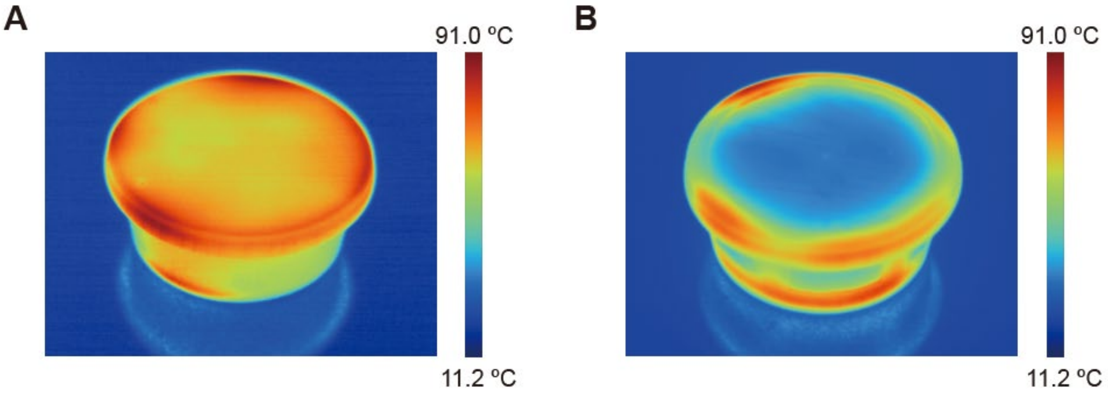

Fig. S19. Thermal imaging after microwave heating. Infrared images of (A) paper bowl and (B) LLM bowl after being heated in a microwave for 1 minute. The paper bowl absorbs microwave energy and its temperature rises, while the LLM bowl does not get heated by microwaves and remains at a lower temperature.

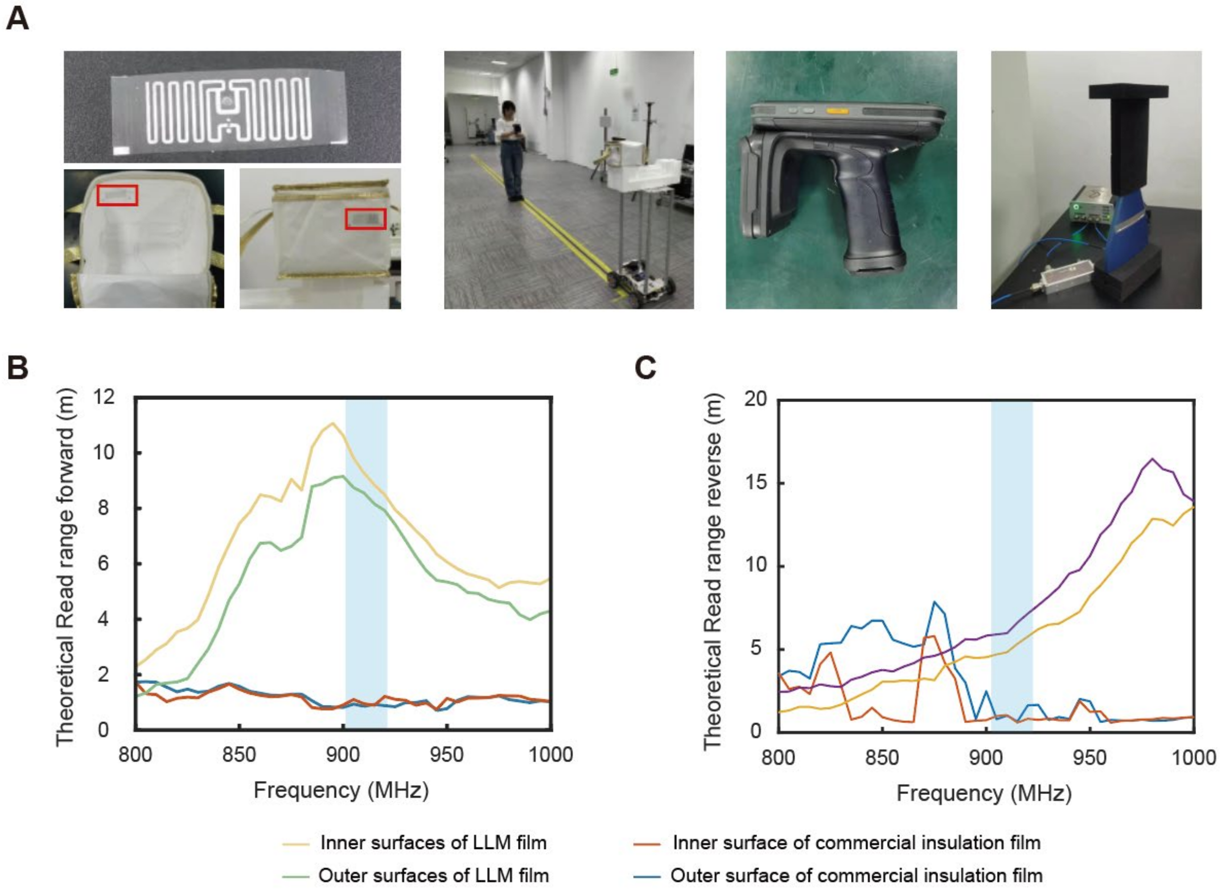

Fig. S20. RFID read-range performance of insulation bags. (A) Photographs showing RFID tags, their positions on the outer and inner surfaces of the insulation bags, the handheld RFID reader, and the theoretical read range test equipment. (B) Measured theoretical forward read ranges of RFID tags. (C) Measured theoretical reverse read ranges of RFID tags. The LLM insulation bag exhibited greater theoretical read ranges, both forward and reverse, compared to the commercial insulation bag. This indicates that LLM can effectively meet the requirements for both thermal insulation performance and RFID scanning performance in smart cold chain transportation.

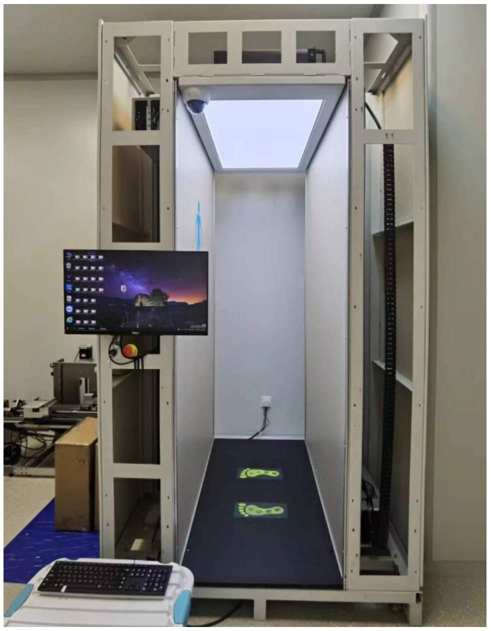

Fig. S21. Photograph of THz security imaging system.

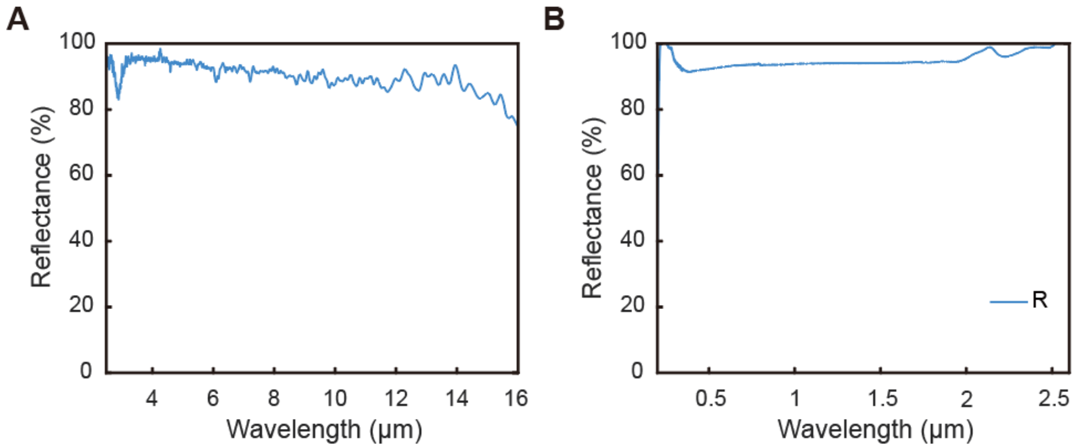

Fig. S22. Optical Properties of Micro-KBr after Sintering. (A) Spectral reflectance of micro-KBr in the mid-infrared (MIR) range (2.5 - 16 μm). (B) Spectral reflectance and transmission of micro-KBr in the visible and NIR wavelength ranges.

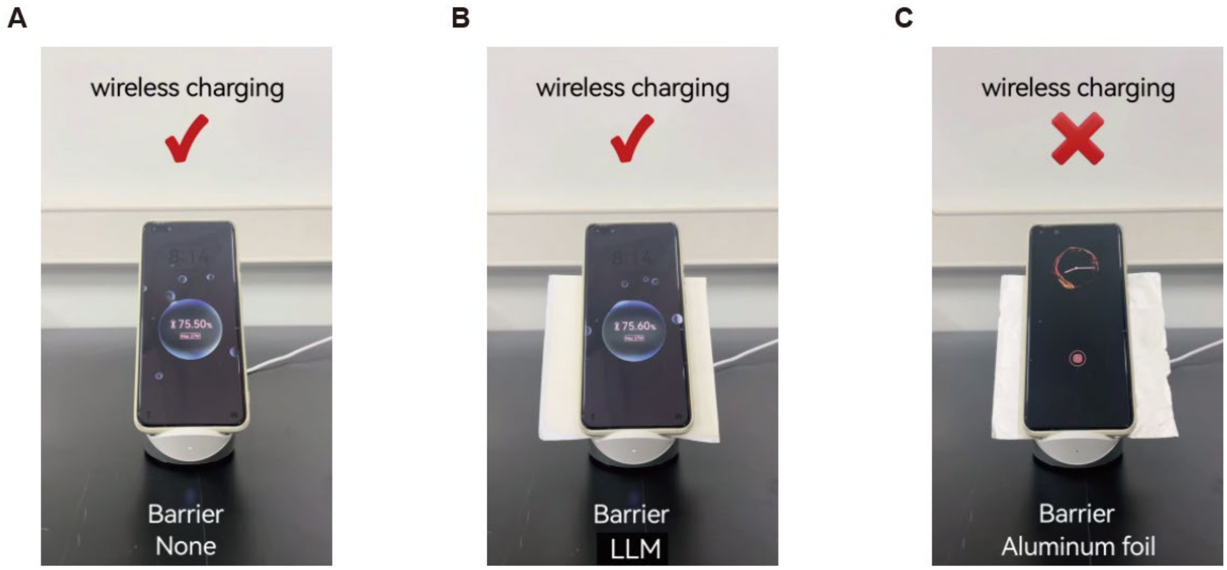

Fig. S23. Wireless-charging demonstration through different barriers. Phone-screen indicators show successful 27 W charging with (A) no barrier or (B) an LLM barrier, but failure when (C) an aluminum-foil barrier is inserted.

Supplementary Movies Captions

Movie S1. Wireless communication experiment

The movie shows a real-time wireless communication experiment at 26 GHz. When an LLM panel is inserted between the transmitter and receiver, the transmitted video plays back continuously without interruption. In contrast, when a commercial metallic low-e insulation wall sticker is inserted, the signal is blocked, causing the video to freeze. This demonstrates the superior longwave (millimeter-wave) transmittance of the LLM, enabling high-quality wireless communication through insulation barriers.

Movie S2. Wireless charging test

The movie demonstrates wireless charging compatibility. A smartphone charges normally at 27W when placed directly on a wireless charger. The charging remains unaffected (27W) when LLM is placed between the phone and the charger. However, charging fails completely when aluminum foil (a metallic low-e material) is inserted instead, highlighting LLM's transparency to wireless power transfer frequencies (110–205 kHz).

Movie S3. Wireless doorbell test

The movie shows a wireless doorbell test. When the doorbell (447 MHz) is enclosed inside a foam box lined with LLM on all six inner walls, pressing the remote switch outside successfully triggers the doorbell, proving signal penetration. Conversely, when the box is lined with aluminum foil, the wireless signal is fully shielded, and the doorbell does not ring, demonstrating LLM’s compatibility with common wireless control signals in building environments.

REFERENCES

1. F. Nicholas, W. Weimin, Alvine Kyle J., K. Srinivas, "*Energy Savings Potential of Radiative Cooling Technologies*" (PNNL--24904, 1234791, 2015).

2. "*The Future of Cooling: Opportunities for Energy-efficient Air Conditioning*" (International Energy Agency, 2018).

3. S. Bouckaert, A. F. Pales, C. McGlade, U. Remme, B. Wanner, L. Varro, D. D'Ambrosio, T. Spencer, "Net zero by 2050: A roadmap for the global energy sector" (International Energy Agency, 2021) https://iea.org/reports/net-zero-by-2050.

4. S. Fan, W. Li, Photonics and thermodynamics concepts in radiative cooling. *Nat. Photonics* **16**, 182–190 (2022).

5. X. Yin, R. Yang, G. Tan, S. Fan, Terrestrial radiative cooling: Using the cold universe as a renewable and sustainable energy source. *Science* **370**, 786–791 (2020).

6. A. P. Raman, M. A. Anoma, L. Zhu, E. Rephaeli, S. Fan, Passive radiative cooling below ambient air temperature under direct sunlight. *Nature* **515**, 540–544 (2014).

7. Y. Zhai, Y. Ma, S. N. David, D. Zhao, R. Lou, G. Tan, R. Yang, X. Yin, Scalable-manufactured randomized glass-polymer hybrid metamaterial for daytime radiative cooling. *Science* **355**, 1062–1066 (2017).

8. J. Mandal, Y. Fu, A. C. Overvig, M. Jia, K. Sun, N. N. Shi, H. Zhou, X. Xiao, N. Yu, Y. Yang, Hierarchically porous polymer coatings for highly efficient passive daytime radiative cooling. *Science* **362**, 315–319 (2018).

9. T. Li, Y. Zhai, S. He, W. Gan, Z. Wei, M. Heidarinejad, D. Dalgo, R. Mi, X. Zhao, J. Song, J. Dai, C. Chen, A. Aili, A. Vellore, A. Martini, R. Yang, J. Srebric, X. Yin, L. Hu, A radiative cooling structural material. *Science* **364**, 760–763 (2019).

10. D. Li, X. Liu, W. Li, Z. Lin, B. Zhu, Z. Li, J. Li, B. Li, S. Fan, J. Xie, J. Zhu, Scalable and hierarchically designed polymer film as a selective thermal emitter for high-performance all-day radiative cooling. *Nat. Nanotechnol.* **16**, 153–158 (2021).

11. B. Zhu, W. Li, Q. Zhang, D. Li, X. Liu, Y. Wang, N. Xu, Z. Wu, J. Li, X. Li, P. B. Catrysse, W. Xu, S. Fan, J. Zhu, Subambient daytime radiative cooling textile based on nanoprocessed silk. *Nat. Nanotechnol.* **16**, 1342–1348 (2021).

12. K. Lin, S. Chen, Y. Zeng, T. C. Ho, Y. Zhu, X. Wang, F. Liu, B. Huang, C. Y.-H. Chao, Z. Wang, C. Y. Tso, Hierarchically structured passive radiative cooling ceramic with high solar reflectivity. *Science* **382**, 691–697 (2023).

13. X. Zhao, T. Li, H. Xie, H. Liu, L. Wang, Y. Qu, S. C. Li, S. Liu, A. H. Brozena, Z. Yu, J. Srebric, L. Hu, A solution-processed radiative cooling glass. *Science* **382**, 684–691 (2023).

14. F. Xie, W. Jin, J. R. Nolen, H. Pan, N. Yi, Y. An, Z. Zhang, X. Kong, F. Zhu, K. Jiang, S. Tian, T. Liu, X. Sun, L. Li, D. Li, Y.-F. Xiao, A. Alu, S. Fan, W. Li, Subambient daytime radiative cooling of vertical surfaces. *Science* **386**, 788–794 (2024).

15. L. Zhou, J. Rada, H. Zhang, H. Song, S. Mirniaharikandi, B. S. Ooi, Q. Gan, Sustainable and inexpensive polydimethylsiloxane sponges for daytime radiative cooling. *Adv. Sci.* **8**, 2102502 (2021).

16. R. H. Galib, Y. Tian, Y. Lei, S. Dang, X. Li, A. Yudhanto, G. Lubineau, Q. Gan, Atmospheric-moisture-induced polyacrylate hydrogels for hybrid passive cooling. *Nat. Commun.* **14**, 6707 (2023).

17. K.-T. Lin, X. Nian, K. Li, J. Han, N. Zheng, X. Lu, C. Guo, H. Lin, B. Jia, Highly efficient flexible structured metasurface by roll-to-roll printing for diurnal radiative cooling. *eLight* **3**, 22 (2023).

18. Z. Fang, N. Li, B. Li, G. Luo, Y. Huang, The effect of building envelope insulation on cooling energy consumption in summer. *Energ. Buildings* **77**, 197–205 (2014).

19. B. P. Jelle, S. E. Kalnæs, T. Gao, Low-emissivity materials for building applications: A state-of-the-art review and future research perspectives. *Energ. Buildings* **96**, 329–356 (2015).

20. L. Belussi, B. Barozzi, A. Bellazzi, L. Danza, A. Devitofrancesco, C. Fanciulli, M. Ghellere, G. Guazzi, I. Meroni, F. Salamone, F. Scamoni, C. Scrosati, A review of performance of zero energy buildings and energy efficiency solutions. *J. Build. Eng.* **25**, 100772 (2019).

21. Y. Peng, Y. Cui, Advanced textiles for personal thermal management and energy. *Joule* **4**, 724–742 (2020).

22. J. Xu, A. P. Raman, Controlling radiative heat flows in interior spaces to improve heating and cooling efficiency. *iScience* **24**, 102825 (2021).

23. Y. Zhu, Y. Zhou, B. Qin, R. Qin, M. Qiu, Q. Li, Night-time radiative warming using the atmosphere. *Light Sci. Appl.* **12**, 268 (2023).

24. Y. Peng, L. Fan, W. Jin, Y. Ye, Z. Huang, S. Zhai, X. Luo, Y. Ma, J. Tang, J. Zhou, L. C. Greenburg, A. Majumdar, S. Fan, Y. Cui, Coloured low-emissivity films for building envelopes for year-round energy savings. *Nat. Sustainability* **5**, 339–347 (2022).

25. A. Joudi, H. Svedung, C. Bales, M. Rönnelid, Highly reflective coatings for interior and exterior steel cladding and the energy efficiency of buildings. *Appl. Energy* **88**, 4655–4666 (2011).

26. M. J. Tenpierik, E. Hasselaar, Reflective multi-foil insulations for buildings: A review. *Energ. Buildings* **56**, 233–243 (2013).

27. Y. Peng, J.-C. Lai, X. Xiao, W. Jin, J. Zhou, Y. Yang, X. Gao, J. Tang, L. Fan, S. Fan, Z. Bao, Y. Cui, Colorful low-emissivity paints for space heating and cooling energy savings. *Proc. Natl. Acad. Sci. U.S.A.* **120**, e2300856120 (2023).

28. R. Yin, P. Xu, P. Shen, Case study: Energy savings from solar window film in two commercial buildings in Shanghai. *Energ. Buildings* **45**, 132–140 (2012).

29. K. Tang, K. Dong, J. Li, M. P. Gordon, F. G. Reichertz, H. Kim, Y. Rho, Q. Wang, C.-Y. Lin, C. P. Grigoropoulos, A. Javey, J. J. Urban, J. Yao, R. Levinson, J. Wu, Temperature-adaptive radiative coating for all-season household thermal regulation. *Science* **374**, 1504–1509 (2021).

30. S. Wang, T. Jiang, Y. Meng, R. Yang, G. Tan, Y. Long, Scalable thermochromic smart windows with passive radiative cooling regulation. *Science* **374**, 1501–1504 (2021).

31. D. Videira-Quintela, O. Martin, G. Montalvo, Recent advances in polymer-metallic composites for food packaging applications. *Trends Food Sci. Technol.* **109**, 230–244 (2021).

32. S. Singh, G. Burgess, J. Singh, Performance comparison of thermal insulated packaging boxes, bags and refrigerants for single-parcel shipments. *Packag. Technol. Sci.* **21**, 25–35 (2008).

33. P.-C. Hsu, C. Liu, A. Y. Song, Z. Zhang, Y. Peng, J. Xie, K. Liu, C.-L. Wu, P. B. Catrysse, L. Cai, S. Zhai, A. Majumdar, S. Fan, Y. Cui, A dual-mode textile for human body radiative heating and cooling. *Sci. Adv.* **3**, e1700895 (2017).

34. H. Luo, Q. Li, K. Du, Z. Xu, H. Zhu, D. Liu, L. Cai, P. Ghosh, M. Qiu, An ultra-thin colored textile with simultaneous solar and passive heating abilities. *Nano Energy* **65**, 103998 (2019).

35. C. Xu, G. T. Stiubianu, A. A. Gorodetsky, Adaptive infrared-reflecting systems inspired by cephalopods. *Science* **359**, 1495–1500 (2018).

36. B. Song, D. Thompson, A. Fiorino, Y. Ganjeh, P. Reddy, E. Meyhofer, Radiative heat conductances between dielectric and metallic parallel plates with nanoscale gaps. *Nat. Nanotechnol.* **11**, 509–514 (2016).

37. P.-C. Hsu, X. Liu, C. Liu, X. Xie, H. R. Lee, A. J. Welch, T. Zhao, Y. Cui, Personal thermal management by metallic nanowire-coated textile. *Nano Lett.* **15**, 365–371 (2015).

38. M. Shi, M. Shen, X. Guo, X. Jin, Y. Cao, Y. Yang, W. Wang, J. Wang, Ti3C2Tx MXene-decorated nanoporous polyethylene textile for passive and active personal precision heating. *ACS Nano* **15**, 11396–11405 (2021).

39. E. M. Leung, M. Colorado Escobar, G. T. Stiubianu, S. R. Jim, A. L. Vyatskikh, Z. Feng, N. Garner, P. Patel, K. L. Naughton, M. Follador, E. Karshalev, M. D. Trexler, A. A. Gorodetsky, A dynamic thermoregulatory material inspired by squid skin. *Nat. Commun.* **10**, 1947 (2019).

40. M. Born, E. Wolf, *Principles of optics: Electromagnetic Theory of Propagation, Interference and Diffraction of Light*. (Elsevier, 2013).

41. I. Rodriguez, H. C. Nguyen, N. T. Jorgensen, T. B. Sorensen, P. Mogensen, Radio propagation into modern buildings: Attenuation measurements in the range from 800 MHz to 18 GHz, in proceedings of the *2014 IEEE 80th Vehicular Technology Conference* (VTC2014-Fall) (IEEE, 2014), 1–5.

42. P. Ragulis, P. Ängskog, R. Simniškis, B. Vallhagen, M. Bäckström, Ž. Kancleris, Shielding effectiveness of modern energy-saving glasses and windows. *IEEE Trans. Antennas Propag.* **65**, 4250–4258 (2017).

43. A. Karttunen, M. Mökkönen, K. Haneda, Investigation of 5G radio frequency signal losses of glazing structures, in *proceedings of the All Eyes on Smarter Glass Performance Day 2019* (Glass performance days, 2019), 13–17.

44. L. Yunos, M. L. Jane, P. J. Murphy, K. Zuber, Frequency selective surface on low emissivity windows as a means of improving telecommunication signal transmission: A review. *J. Build. Eng.* **70**, 106416 (2023).

45. "Mobile network reception problems in low energy buildings - Working group report" (Ministry of Transport and Communications Working Group, 2013) https://julkaisut.valtioneuvosto.fi/handle/10024/77948.

46. W. B. Kuhn, Wireless communication problems in energy-efficient building construction,in proceedings of the *2016 IEEE International Symposium on Electromagnetic Compatibility (EMC)* (IEEE, 2016), pp. 857–861.

47. “The Effect of Building Materials on Indoor Mobile Performance” (Commission for Communications Regulation, 2018) https://comreg.ie/?dlm_download=the-effect-of-building-materials-on-indoor-mobile-performance.

48. L. Ruiz-Garcia, L. Lunadei, in *Sustainable Radio Frequency Identification Solutions*. (2010), vol. 2, pp. 37–50.

49. G. Tagliabue, H. A. Atwater, A. Polman, E. Cortés, Photonic solutions help fight climate crisis. *Nat. Photonics* **18**, 879–882 (2024).

50. R. J. W. Brienen, O. L. Phillips, T. R. Feldpausch, E. Gloor, T. R. Baker, J. Lloyd, G. Lopez-Gonzalez, A. Monteagudo-Mendoza, Y. Malhi, S. L. Lewis, R. V. Martinez, M. Alexiades, E. Á. Dávila, P. Alvarez-Loayza, A. Andrade, L. E. O. C. Aragão, A. Araujo-Murakami, E. J. M. M. Arets, L. Arroyo, G. A. Aymard C, O. S. Bánki, C. Baraloto, J. Barroso, D. Bonal, R. G. A. Boot, J. L. C. Camargo, C. V. Castilho, V. Chama, K. J. Chao, J. Chave, J. A. Comiskey, F. C. Valverde, L. da Costa, E. A. de Oliveira, A. Di Fiore, T. L. Erwin, S. Fauset, M. Forsthofer, D. R. Galbraith, E. S. Grahame, N. Groot, B. Hérault, N. Higuchi, E. N. H. Coronado, H. Keeling, T. J. Killeen, W. F. Laurance, S. Laurance, J. Licona, W. E. Magnussen, B. S. Marimon, B. H. Marimon-Junior, C. Mendoza, D. A. Neill, E. M. Nogueira, P. Núñez, N. C. P. Camacho, A. Parada, G. Pardo-Molina, J. Peacock, M. Peña-Claros, G. C. Pickavance, N. C. A. Pitman, L. Poorter, A. Prieto, C. A. Quesada, F. Ramírez, H. Ramírez-Angulo, Z. Restrepo, A. Roopsind, A. Rudas, R. P. Salomão, M. Schwarz, N. Silva, J. E. Silva-Espejo, M. Silveira, J. Stropp, J. Talbot, H. ter Steege, J. Teran-Aguilar, J. Terborgh, R. Thomas-Caesar, M. Toledo, M. Torello-Raventos, R. K. Umetsu, G. M. F. van der Heijden, P. van der Hout, I. C. G. Vieira, S. A. Vieira, E. Vilanova, V. A. Vos, R. J. Zagt, Long-term decline of the Amazon carbon sink. *Nature* **519**, 344–348 (2015).

51. M. R. Querry, “Optical constants of minerals and other materials from the millimeter to the ultraviolet” (Chemical Research, Development & Engineering Center, US Army Armament Munitions Cchemical Command, 1987). https://lweb.cfa.harvard.edu/HITRAN/HITRAN2012/Aerosols/papers/querry_minerals_1987.pdf.

52. J. E. Eldridge, E. D. Palik, in *Handbook of Optical Constants of Solids*, E. D. Palik, Ed. (Academic Press, 1997), pp. 775–793.

53. M. Z. M. Ashhar, L. C. Haw, Recent research and development on the use of reflective technology in buildings–A review. *J. Build. Eng.* **45**, 103552 (2022).

54. H. H. Li, Refractive index of alkali halides and its wavelength and temperature derivatives. *J. Phys. Chem. Ref. Data Monogr.* **5**, 329–528 (1976).

55. M. N. Afsar, K. J. Button, Millimeter-wave dielectric measurement of materials. *Proc. IEEE* **73**, 131–153 (1985).

56. L. Liu, H. Chang, T. Xu, Y. Song, C. Zhang, Z. H. Hang, X. Hu, Achieving low-emissivity materials with high transmission for broadband radio-frequency signals. *Sci. Rep.* **7**, 4840 (2017).

57. N. Soares, J. J. Costa, A. R. Gaspar, P. Santos, Review of passive PCM latent heat thermal energy storage systems towards buildings' energy efficiency. *Energ. Buildings* **59**, 82–103 (2013).

58. S. K. Agrawal, K. Sharma, 5G millimeter wave (mmWave) communications, in proceedings of the *2016 3rd International Conference on Computing for Sustainable Global Development (INDIACom)* (IEEE, 2016), 3630–3634.

59. K. Leng, L. Jin, W. Shi, I. Van Nieuwenhuyse, Research on agricultural products supply chain inspection system based on internet of things. *Clust Comput* **22**, 8919–8927 (2019).

60. R. Badia-Melis, U. Mc Carthy, L. Ruiz-Garcia, J. Garcia-Hierro, J. R. Villalba, New trends in cold chain monitoring applications-A review. *Food Control* **86**, 170–182 (2018).

61. J. F. Federici, B. Schulkin, F. Huang, D. Gary, R. Barat, F. Oliveira, D. Zimdars, THz imaging and sensing for security applications—explosives, weapons and drugs. *Semicond. Sci. Technol.* **20**, S266–S280 (2005).

62. K. Kawase, Y. Ogawa, Y. Watanabe, H. Inoue, Non-destructive terahertz imaging of illicit drugs using spectral fingerprints. *Opt. Express* **11**, 2549–2554 (2003).

63. P.-C. Hsu, A. Y. Song, P. B. Catrysse, C. Liu, Y. Peng, J. Xie, S. Fan, Y. Cui, Radiative human body cooling by nanoporous polyethylene textile. *Science* **353**, 1019–1023 (2016).

64. L. Cai, A. Y. Song, P. Wu, P.-C. Hsu, Y. Peng, J. Chen, C. Liu, P. B. Catrysse, Y. Liu, A. Yang, C. Zhou, C. Zhou, S. Fan, Y. Cui, Warming up human body by nanoporous metallized polyethylene textile. *Nat. Commun.* **8**, 496 (2017).

65. S. Zeng, S. Pian, M. Su, Z. Wang, M. Wu, X. Liu, M. Chen, Y. Xiang, J. Wu, M. Zhang, Q. Cen, Y. Tang, X. Zhou, Z. Huang, R. Wang, A. Tunuhe, X. Sun, Z. Xia, M. Tian, M. Chen, X. Ma, L. Yang, J. Zhou, H. Zhou, Q. Yang, X. Li, Y. Ma, G. Tao, Hierarchical-morphology metafabric for scalable passive daytime radiative cooling. *Science* **373**, 692–696 (2021).

66. Y. Peng, J. Chen, A. Y. Song, P. B. Catrysse, P.-C. Hsu, L. Cai, B. Liu, Y. Zhu, G. Zhou, D. S. Wu, H. R. Lee, S. Fan, Y. Cui, Nanoporous polyethylene microfibres for large-scale radiative cooling fabric. *Nat. Sustainability* **1**, 105–112 (2018).

67. M. Guo, Y. Zheng, Q. Chen, L. Ding, D. Sang, F. Yuan, T. Guo, Y. Fu, Analysis and design of a high-transmittance performance for varactor-tunable frequency-selective surface. *IEEE Trans. Antennas Propag.* **69**, 4623–4632 (2021).

68. J. Y. Dai, W. Tang, L. X. Yang, X. Li, M. Z. Chen, J. C. Ke, Q. Cheng, S. Jin, T. J. Cui, Realization of multi-modulation schemes for wireless communication by time-domain digital coding metasurface. *IEEE Trans. Antennas Propag.* **68**, 1618–1627 (2020).
